# miR-146a targets *Fos* expression in human cardiac cells

**DOI:** 10.1242/dmm.020768

**Published:** 2015-09-01

**Authors:** Xavier Palomer, Eva Capdevila-Busquets, Gaia Botteri, Mercy M. Davidson, Cristina Rodríguez, José Martínez-González, Francisco Vidal, Emma Barroso, Tung O. Chan, Arthur M. Feldman, Manuel Vázquez-Carrera

**Affiliations:** 1 Department of Pharmacology and Therapeutic Chemistry, IBUB (Institut de Biomedicina de la Universitat de Barcelona) and CIBER de Diabetes y Enfermedades Metabólicas Asociadas (CIBERDEM), Faculty of Pharmacy, University of Barcelona, Diagonal 643, Barcelona E-08028, Spain; 2 Department of Radiation Oncology, Columbia University, P&S 11-451, 630 West 168th Street, New York, NY 10032, USA; 3 Centro de Investigación Cardiovascular, CSIC-ICCC, IIB-Sant Pau, Avda. Sant Antoni Maria Claret 167, Barcelona 08025, Spain; 4 Unitat de Diagnòstic i Teràpia Molecular, Banc de Sang i Teixits, Passeig Vall d'Hebron 119-129, Barcelona 08035, Spain; 5 Department of Medicine, The Center for Translational Medicine, Jefferson Medical College, 1025 Walnut Street, Philadelphia, PA 19107, USA; 6 Departments of Medicine and Physiology, Cardiovascular Research Center, Temple University School of Medicine, 3500 N, Broad Street, Philadelphia, PA 19140, USA

**Keywords:** Fos, Cardiac remodeling, Inflammation, miR-146a, Matrix metalloproteinase-9

## Abstract

miR-146a is a microRNA whose transcript levels are induced in the heart upon activation of NF-κB, a transcription factor induced by pro-inflammatory molecules (such as TNF-α) that is strongly related to the pathogenesis of cardiac disorders. The main goal of this study consisted of studying new roles of miR-146a in cardiac pathological processes caused by the pro-inflammatory cytokine TNF-α. Our results demonstrate that miR-146a transcript levels were sharply increased in cardiac ventricular tissue of transgenic mice with specific overexpression of TNF-α in the heart, and also in a cardiomyocyte cell line of human origin (AC16) exposed to TNF-α. Among all the *in silico* predicted miR-146a target genes, Fos mRNA and protein levels notably decreased after TNF-α treatment or miR-146a overexpression. These changes correlated with a diminution in the DNA-binding activity of AP-1, the Fos-containing transcription factor complex. Interestingly, AP-1 inhibition was accompanied by a reduction in matrix metalloproteinase (*MMP*)*-9* mRNA levels in human cardiac cells. The specific regulation of this MMP by miR-146a was further confirmed at the secretion and enzymatic activity levels, as well as after anti-miR-mediated miR-146a inhibition. The results reported here demonstrate that *Fos* is a direct target of miR-146a activity and that downregulation of the Fos–AP-1 pathway by miR-146a has the capacity to inhibit MMP-9 activity. Given that *MMP-9* is an AP-1 target gene involved in cardiac remodeling, myocardial dysfunction and progression of heart failure, these findings suggest that miR-146a might be a new and promising therapeutic tool for treating cardiac disorders associated with enhanced inflammation in the heart.

## Abstract (precis)

**Summary:** These findings demonstrate that Fos is a direct target of miR-146a activity and that downregulation of the Fos–AP-1 pathway by miR-146a can inhibit MMP-9 activity.

## INTRODUCTION

The myocardium responds to various pathological stimuli by expressing and secreting several pro-inflammatory cytokines and chemokines such as interleukin-6 (IL-6), monocyte chemoattractant protein-1 (MCP-1) and tumor necrosis factor-α (TNF-α) (Palomer et al., 2013a,b). These pro-inflammatory mediators, which are transcriptionally regulated by the ubiquitous and inducible nuclear factor-κB (NF-κB), exert their pleiotropic autocrine effects via downstream activation of activator protein-1 (AP-1) and NF-κB itself, thereby contributing to myocardial inflammation, dilated cardiomyopathy, cardiac hypertrophy and heart failure (Gupta et al., 2008; Palomer et al., 2013a,b). Myocardial injury caused by these pathologies leads to myocyte function failure, fibrosis and ensuing ventricular remodeling, which can eventually trigger heart failure. In this regard, TNF-α production is enhanced in the heart of spontaneously hypertensive rats and in the failing human heart; this then contributes to cardiac remodeling and malfunction, thereby speeding up heart failure progression (Bergman et al., 1999). Likewise, continual intra-cardiac expression of TNF-α promotes the development of cardiac allograft hypertrophy (Stetson et al., 2001). Therefore, it is not surprising that pharmacological inhibition of TNF-α activity improves myocardial function during heart failure (Isic et al., 2008).

A recent study performed in rats has revealed an essential role for NF-κB and AP-1 in the pathogenesis of cardiac hypertrophy (Wang et al., 2009). AP-1 is a heterodimeric transcription factor composed of members belonging to the Jun, Fos, activating transcription factors (ATFs) and Jun-dimerization partner families. AP-1 regulates a number of cellular processes, including differentiation, proliferation and apoptosis. In the heart, AP-1 causes changes in the extracellular matrix and decreases contractility and cell permeability, inducing hypertrophy of cardiomyocytes and fibrosis of the interstitial substance, which eventually lead to heart failure (Wang et al., 2009).

The last decade has seen a plethora of studies reporting a critical role of microRNAs (miRNAs) as regulators of cardiac development, function and performance, as well as in the development of cardiac disease. miRNAs are endogenous non-coding small RNAs that modulate gene expression by targeting mRNAs for post-transcriptional repression through imperfect base-pairing, thereby participating in many essential biological processes. To date, more than 900 mature miRNAs have been identified in the human genome, which alter the translation of up to 50% of all genes (van de Vrie et al., 2011). Furthermore, a single miRNA could target multiple mRNAs, and most mRNAs can be regulated by different miRNAs, hence bringing enormous complexity to the regulation of gene expression. Chronic immune activation and anomalous miRNA expression have been regarded as important players in the pathological molecular mechanisms underlying cardiac disease. As an example, microarray studies have shed some light on the specific miRNAs that are aberrantly downregulated (miR-1, -29, -30, -133 and -150) or upregulated (miR-21, -23a, -125, -146, -195, -199 and -214) in heart failure patients (van de Vrie et al., 2011). However, little is known at present about the function of specific miRNAs during inflammatory responses in the heart. For this reason, the main goal of the present study was to investigate the potential role of miRNAs in the pathological processes induced by TNF-α in cardiac cells.

**TRANSLATIONAL IMPACT**

**Clinical issue**

Heart disease is the leading cause of death in diabetic patients. Diabetic cardiomyopathy is characterized by concentric left ventricular hypertrophy, dilated cardiomyopathy, myocardial fibrosis and subsequent ventricular remodeling, eventually leading to heart failure. However, despite the added burden that diabetes poses on the heart, current therapeutic strategies do not specifically address diabetic cardiomyopathy. An increasing body of evidence suggests a potential link between chronic low-grade inflammation and metabolic disorders such as insulin resistance and type 2 diabetes. Chronic immune activation and aberrant microRNA (miRNA) expression have been regarded as important players in the pathological molecular mechanisms underlying cardiac disease, although little is known at present about the function of specific miRNAs during inflammatory responses in the heart.

**Results**

In this study, the authors investigate the role of miR-146a in the pathological processes induced by the pro-inflammatory cytokine TNF-α in the heart. They find a huge increase in miR-146a levels in the heart of transgenic mice with cardiac-specific overexpression of TNF-α and in human cardiac AC16 cells exposed to TNF-α. The authors demonstrate that *Fos* is a direct target of miR-146a activity: overexpression of the latter results in a notable decrease in both Fos mRNA and protein levels, which correlated with a diminution in the DNA-binding activity of AP-1, the Fos-containing transcription factor complex. The authors also report that AP-1 inhibition is accompanied by a reduction in matrix metalloproteinase (MMP)-9 secretion and enzymatic activity in human cardiac cells.

**Implications and future directions**

In the heart, AP-1 causes changes in the extracellular matrix and decreases contractility, inducing hypertrophy of cardiomyocytes and fibrosis of the interstitial substance, which ultimately lead to heart failure. The results reported here demonstrate that Fos is a direct target of miR-146a activity and that downregulation of the Fos–AP-1 pathway by miR-146a has the capacity to inhibit MMP-9 activity. These results are very appealing because upregulation of MMP-9 expression correlates fairly well with heart failure, whereas its downregulation suppresses ventricular remodeling, myocardial dysfunction and progression of heart failure. The recently developed antisense-oligonucleotide-mediated knockdown and miRNA overexpression techniques have become very attractive pharmacological strategies in the treatment of cardiovascular disease. In this respect, miR-146a emerges as a new and promising therapeutic tool for preventing cardiac disorders associated with inflammatory states in the heart.

## RESULTS

### TNF-α induces miR-146a expression and reduces Fos in cardiac cells

**Fig. 1.**
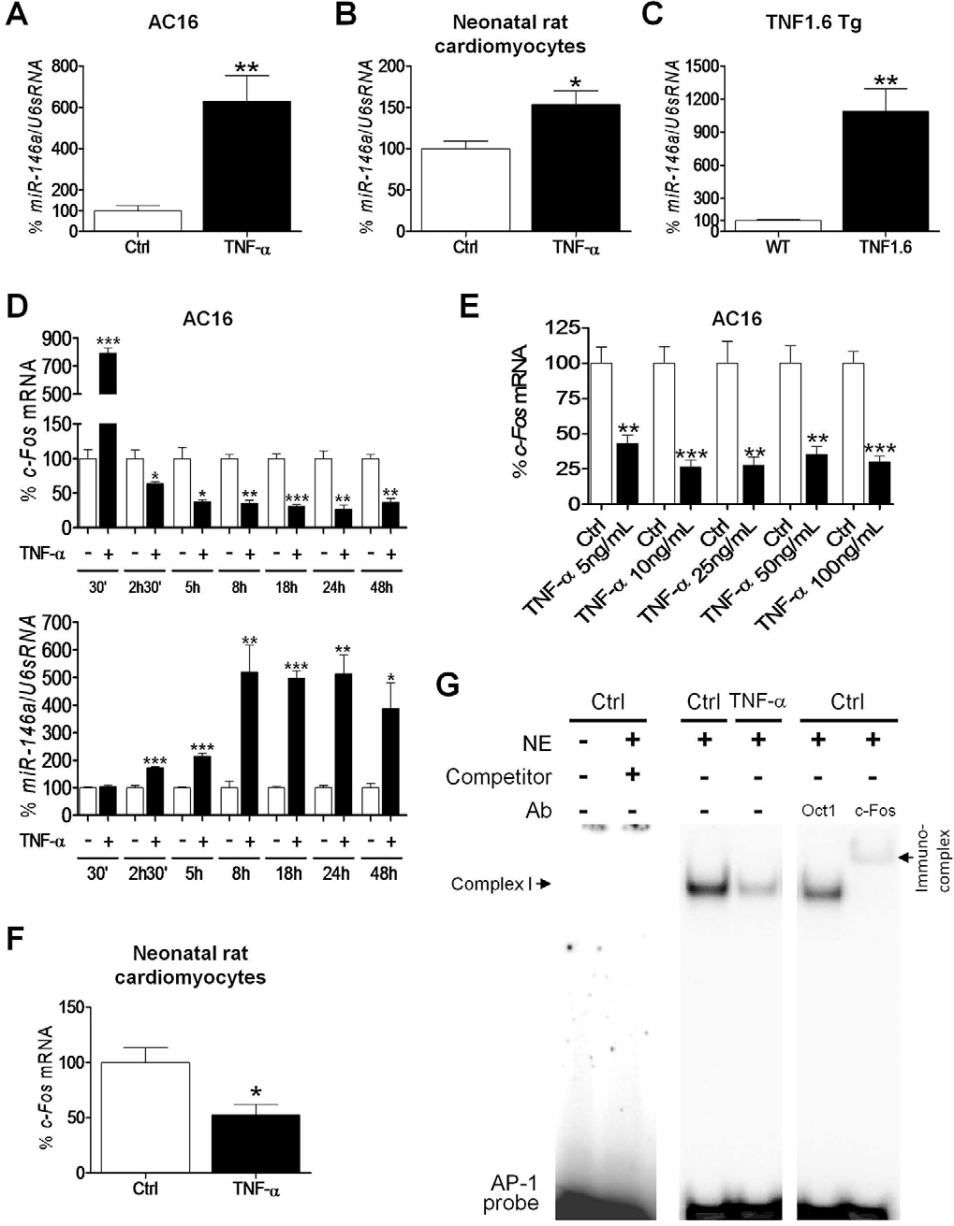
**TNF-α upregulates miR-146a and reduces *Fos* expression in cardiac cells.** Relative quantification of miR-146a levels in samples obtained from: (A) non-differentiated AC16 cells treated with TNF-α (100 ng/ml, 24 h); (B) neonatal rat cardiomyocytes treated with TNF-α (10 ng/ml, 6 h); and (C) left ventricle tissue of transgenic TNF1.6 or control wild-type (WT) mice. Relative quantification of *Fos* and miR-146a expression in: (D) non-differentiated AC16 cells treated with 100 ng/ml TNF-α for 30 min to 48 h; (E) non-differentiated AC16 cells treated with 5, 10, 25, 50 and 100 ng/ml TNF-α for 24 h; and (F) neonatal rat cardiomyocytes treated with TNF-α (10 ng/ml, 6 h). Graphs represent the quantification of (A-C) U6sRNA-, (D,E) 18S- or (F) APRT-normalized mRNA levels, expressed as a percentage of control (Ctrl) or wild-type (WT) samples ±s.d. *P<0.05, **P<0.01 and ***P<0.001 vs Ctrl. (G) EMSA assay showing AP-1 DNA-binding activity in non-differentiated AC16 cells treated with TNF-α. Ab, antibody; NE, nuclear extracts.

As a first approach, we assessed the effects of TNF-α on the expression of a panel of miRNAs previously related to heart disease, obesity, type 2 diabetes and inflammation. Of these, only miR-146a expression was significantly induced by TNF-α [approximately sixfold, *P*<0.01 vs control (Ctrl), Fig. 1A] in human cardiac AC16 cells, whereas the remaining miRNAs were not modified or simply not detected (see supplementary material Fig. S1). To further confirm these results, neonatal rat cardiomyocytes were cultured *in vitro* and treated with TNF-α; as shown in Fig. 1B, miR-146a was also significantly upregulated by this pro-inflammatory cytokine (1.5-fold, *P*<0.05 vs Ctrl). Consistent with this finding, miR-146a was also hugely stimulated in left ventricular tissue of TNF1.6 transgenic mice with cardiac-specific TNF-α overexpression [tenfold, *P*<0.01 vs wild type (WT), Fig. 1C].

The miRGator analysis tool (available at http://genome.ewha.ac.kr/miRGator/miRNAexpression.html), an online interface that uses multiple target prediction algorithms, was then used to identify unknown downstream targets of miR-146a. Of all the predicted genes, *Fos* (FBJ murine osteosarcoma viral oncogene homolog; also known as *c-Fos*) displayed a notable decrease in its mRNA levels in human cardiac AC16 cells after TNF-α treatment, which was sustained over time and with different TNF-α concentrations (Fig. 1D,E). Interestingly, there was a time-dependent significant negative correlation between *Fos* mRNA levels and miR-146a expression in human cardiac AC16 cells (Fig. 1D, Spearman rank correlation *r*=−0.8929, *P*<0.05).

After that, we examined the effects of TNF-α on neonatal rat cardiomyocytes cultured *in vitro* and, in agreement with the enhanced miR-146a levels, TNF-α partially inhibited *Fos* expression (∼50% reduction, *P*<0.05 vs Ctrl, Fig. 1F). Fos is a leucine zipper protein that forms the transcription factor AP-1 and, for this reason, we next carried out an electrophoretic mobility shift assay (EMSA) to assess the transcriptional activity of AP-1. AP-1 formed one specific DNA-binding complex (I) with nuclear proteins in human cardiac cells (Fig. 1G). The competitor lane demonstrated that this complex was specific for the AP-1 probe, whereas addition of a Fos-specific antibody to the binding reaction disrupted the protein:DNA interaction, hence suggesting that complex I contained the Fos subunit. More importantly, treatment of AC16 cells with TNF-α inhibited AP-1 DNA-binding activity compared with control cells.

### miR-146a is directly responsible for Fos downregulation in cardiac cells

**Fig. 2.**
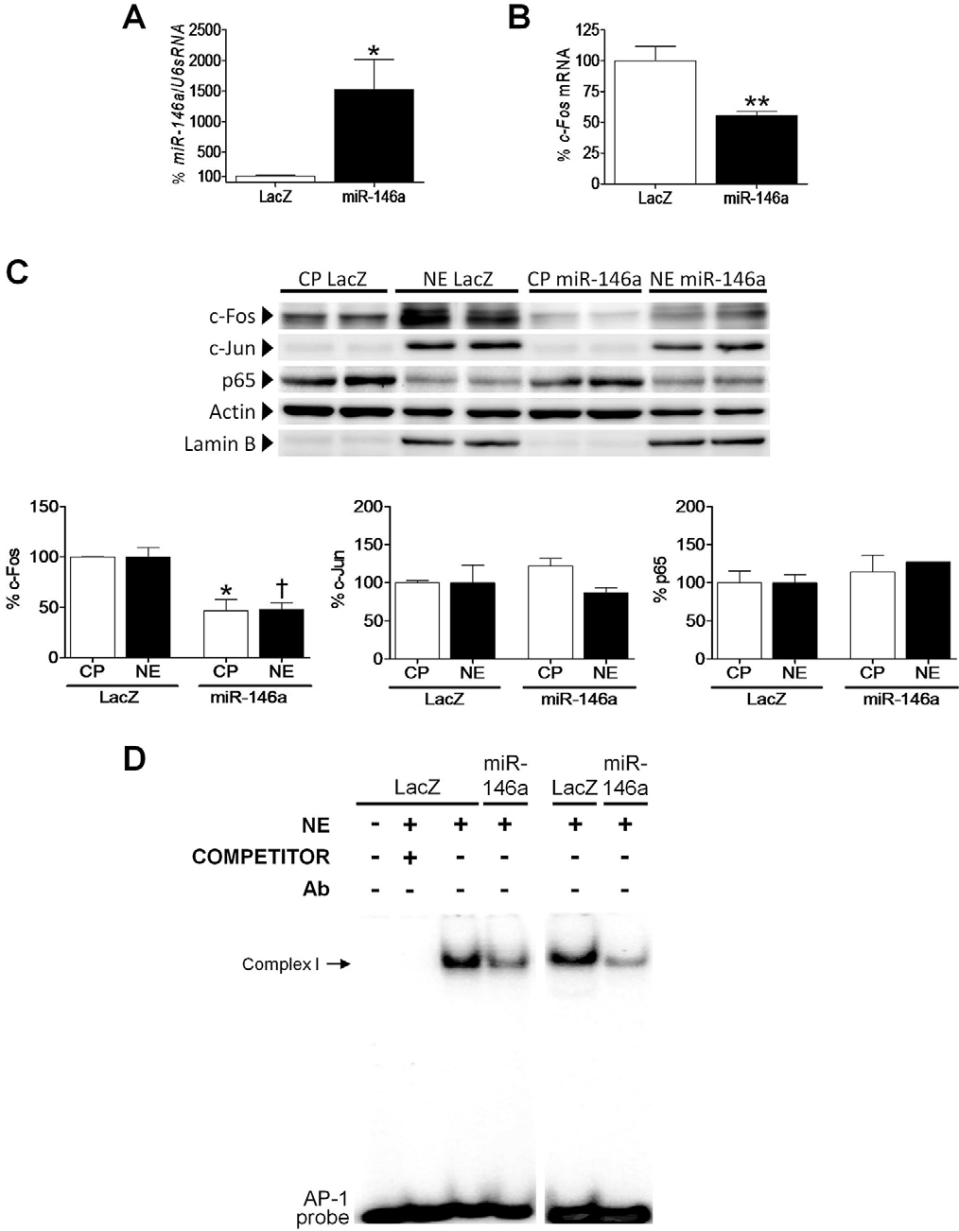
**miR-146a overexpression inhibits Fos in human cardiac cells.** Relative quantification of miR-146a (A) and *Fos* (B) mRNA levels in human cardiac AC16 cells transfected with *lacZ*- or miR-146a-carrying plasmids. The graph represents the quantification of (A) U6sRNA- or (B) 18S-normalized mRNA levels, expressed as a percentage of control samples ±s.d. *P<0.05 and **P<0.01 vs *lacZ*. (C) Western-blot analysis showing the protein levels of Fos, Jun and p65 in cytosolic (CP) and nuclear (NE) protein fractions obtained from human cardiac AC16 cells as described in panel A. To show equal loading of protein, the actin and lamin B signals from the same blot are included. The graphs at the bottom of panel C represent the quantification of protein levels normalized to actin (CP) or lamin B (NE), expressed as a percentage of CP or NE control samples ±s.d. The blot data are representative of two separate experiments. *P<0.05 vs *lacZ* CP; ^†^*P*<0.05 vs *lacZ* NE. (D) EMSA assay showing AP-1 DNA-binding activity after transfection of AC16 cells as described in panel A. Ab, antibody; NE, nuclear extracts.

**Fig. 3.**
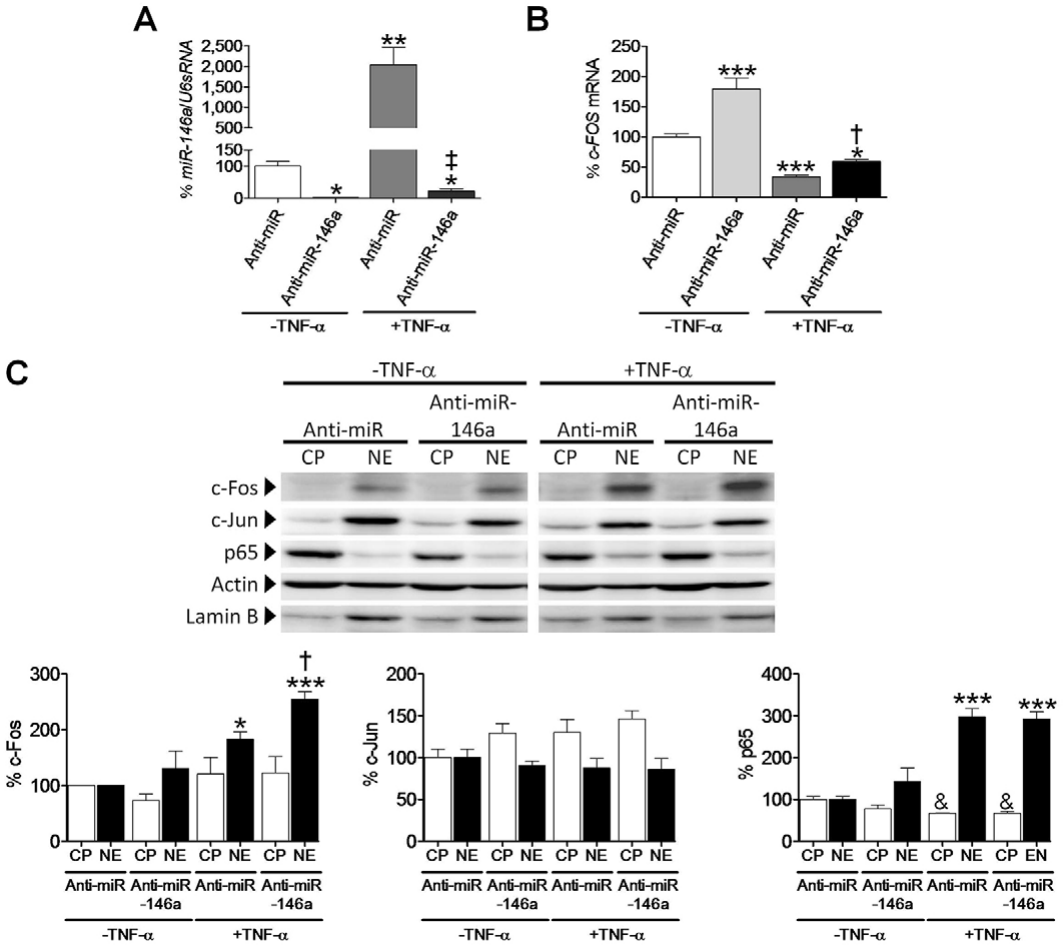
**miR-146a inhibition induces Fos in human cardiac cells.** Relative quantification of miR-146a (A) and *Fos* (B) mRNA levels in human cardiac AC16 cells after transfection with a human anti-miR-146a inhibitor or an anti-miR negative control (Anti-miR). The graph represents the quantification of (A) U6sRNA- or (B) 18S-normalized mRNA levels, expressed as a percentage of control samples ±s.d. *P<0.05, **P<0.01 and ***P<0.001 vs Anti-miR–TNF-α; ^†^*P*<0.05 and ^‡^*P*<0.001 vs Anti-miR+TNF-α. (C) Western-blot analysis showing the protein levels of Fos, Jun and p65 in cytosolic (CP) and nuclear (NE) protein fractions obtained from human cardiac AC16 cells as described in panel A. To show equal loading of protein, the actin and lamin B signals from the same blot are included. The graphs at the bottom of panel C represent the quantification of protein levels normalized to actin (CP) or lamin B (NE), expressed as a percentage of CP or NE control samples ±s.d. The blot data are representative of two separate experiments. *P<0.05 and ***P<0.001 vs NE Anti-miR–TNF-α; ^†^*P*<0.05 vs NE Anti-miR+TNF-α; ^&^*P*<0.05 vs CP Anti-miR–TNF-α.

To determine whether *Fos* was a direct target of miR-146a activity, human AC16 cardiac cells were transfected with a plasmid carrying pre-miR-146a in the absence of TNF-α, which yielded an important increase in miR-146a expression (15-fold, *P*<0.05 vs *lacZ*, Fig. 2A). Compatible with our hypothesis, real-time RT-PCR analysis revealed that this miRNA was inhibiting *Fos* expression (∼45% reduction, *P*<0.01, Fig. 2B). Such inhibition resulted in a significant downregulation of Fos protein levels at both the cytoplasm (CP fraction, ∼50% reduction, *P*<0.05) and the nucleus (NE fraction, ∼50% reduction, *P*<0.05) of miR-146a-transfected cells (Fig. 2C), and caused a significant decrease in the DNA-binding activity of the AP-1 transcription factor (Fig. 2D). In agreement with this, downregulation of miR-146a levels (∼98% reduction, Fig. 3A) by transfecting AC16 cells with a human anti-miR-146a inhibitor coincided with a significant increase in *Fos* expression (1.9-fold, *P*<0.001 vs Ctrl anti-miR, Fig. 3B). In addition, the anti-miR-146a inhibitor partially reversed the effects of TNF-α on *Fos* expression (1.7-fold, *P*<0.001 vs anti-miR+TNF-α). In agreement with gene expression data, Fos protein levels were enhanced by 1.4-fold (*P*<0.05 vs Ctrl anti-miR+TNF-α) in the nucleus of AC16 cells transfected with anti-miR-146a (NE fraction, Fig. 3C). In order to demonstrate the specificity of Fos regulation by miR-146a, the protein levels of Jun and the p65 subunit of NF-κB were also determined in cytoplasmic and nuclear extracts of cells overexpressing miR-146a (Fig. 2C) and after downregulation of miR-146a with an anti-miR-146a inhibitor (Fig. 3C). It is worth mentioning that p65 and Jun were not altered after modulation of miR-146a levels. As expected, p65 protein levels were increased in the nucleus after TNF-α treatment, owing to NF-κB activation. In non-stimulated cells, NF-κB remains inactive in the cytoplasm owing to its heterodimerization with the inhibitor protein IκB. In the presence of a stimulus (e.g. TNF-α), the IκB kinase (IKK) complex phosphorylates IκB, which induces ubiquitylation and ensuing proteasome-mediated degradation of the latter. The subsequent release of the NF-κB heterodimer makes possible its translocation to the nucleus, where it can begin the transcription of its target genes.

### miR-146a modulates inflammation in human cardiac cells

After dimerization with Jun family members and by binding to the so-called TPA-responsive elements (TREs; TGAC/GTCA) in the promoter region of target genes, Fos regulates the expression of genes involved in multiple processes, including inflammation, endoplasmic reticulum stress, metabolism, fibrosis, proliferation and survival (Chinenov and Kerppola, 2001; Durchdewald et al., 2009). Dysregulation of Fos has been linked with a variety of pathological conditions. In the heart, for instance, AP-1 stimulates the expression of inflammatory genes, endothelin, fibronectin, matrix metalloproteinases (MMPs), transforming growth factor (TGF)-β and collagen, thereby displaying potent effects on matrix remodeling and favoring cardiac fibroblast proliferation (Wang et al., 2009; Takimoto and Kass, 2007; Pan et al., 2012). Therefore, in order to determine the pathophysiological relevance of AP-1 down-modulation in human cardiac cells, we next analyzed the expression of various genes that had been reportedly demonstrated to be targeted by the AP-1 transcription factor and also be involved in cardiac disease. As shown in supplementary material Fig. S2, no significant variation was observed in the expression of genes such as *ATF4* (activating transcription factor 4), *BiP/GRP78* (binding immunoglobulin protein/glucose-regulated protein 78), endothelin 1 (*ET-1*), fibronectin 1 (*FN1*), lipin 1, lipoprotein lipase (*LPL*), *TGF-β1*, type I collagen or type IV collagen.

**Fig. 4.**
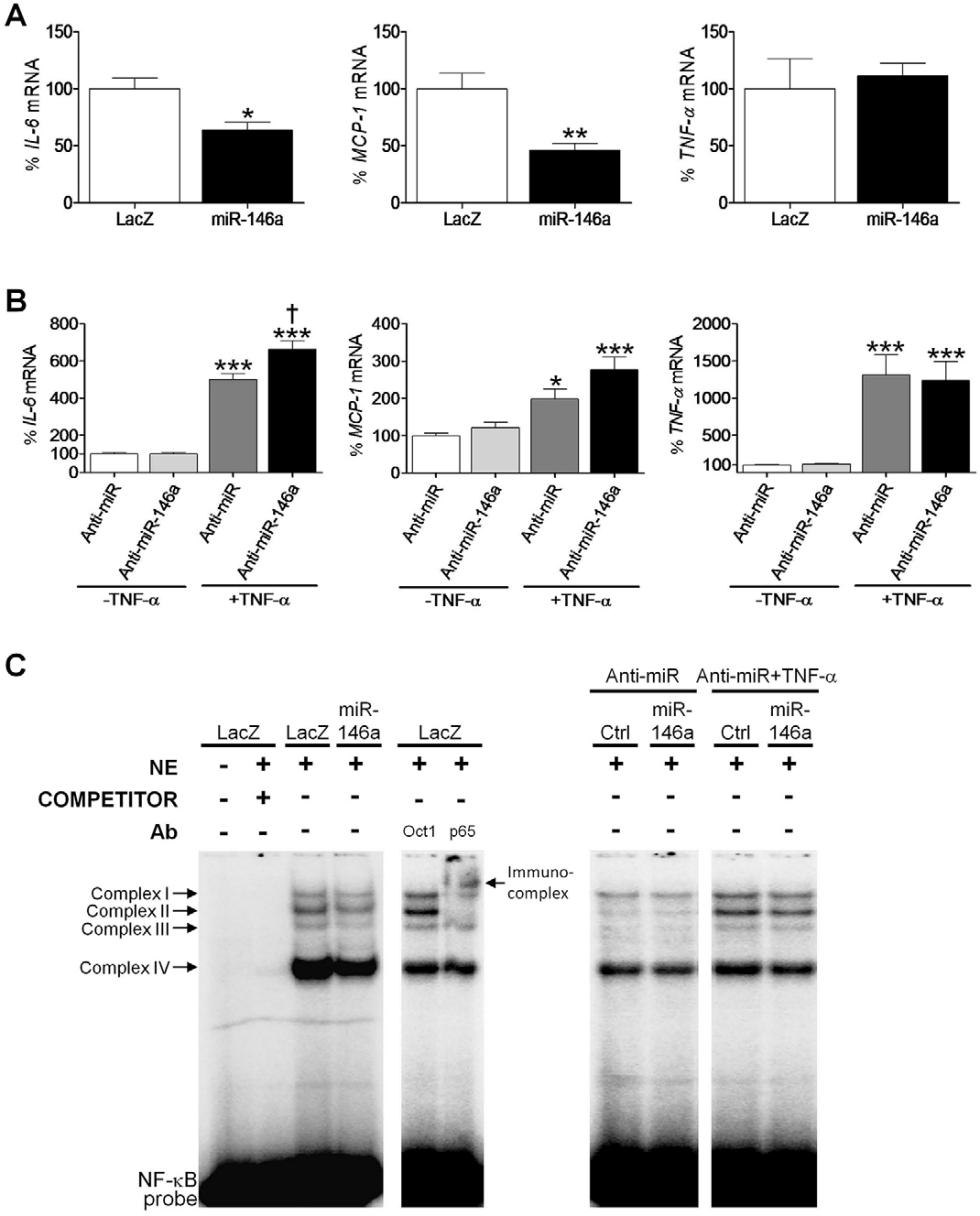
**miR-146a regulates IL-6 and MCP-1 expression in human cardiac cells.** Relative quantification of *IL-6*, *MCP-1* and *TNF-α* mRNA levels in non-differentiated human cardiac AC16 cells transfected with: (A) *lacZ*- or miR-146a-carrying plasmids, or (B) a human anti-miR-146a inhibitor or an anti-miR negative control (Anti-miR). The graphs represent the quantification of 18S-normalized mRNA levels, expressed as a percentage of control samples ±s.d. (A) *P<0.05 and **P<0.01 vs *lacZ*; (B) *P<0.05 and ***P<0.001 vs Anti-miR–TNF-α; ^†^*P*<0.05 vs Anti-miR+TNF-α. (C) EMSA assay showing NF-κB DNA-binding activity after transfection of AC16 cells as described in panels A and B. Ab, antibody; Ctrl, control; NE, nuclear extracts.

On the other hand, miR-146a has been shown to modulate NF-κB activity through the direct targeting of well-established mediators of its activation, including interleukin-1 receptor-associated kinase (IRAK)1, IRAK2, TNF receptor-associated factor (TRAF)2 and TRAF6 (Wang et al., 2013; Tanic et al., 2012). However, none of the transcript levels for these genes was found to be modified after miR-146a overexpression or inhibition, thus indicating that they were not regulated by this miRNA in human cardiac AC16 cells (see supplementary material Fig. S3). However, miR-146a overexpression inhibited *IL-6* (∼40% reduction, *P*<0.05 vs *lacZ*) and *MCP-1* (∼50% reduction, *P*<0.01) transcript levels, although *TNF-α* levels remained unaltered (Fig. 4A). In consonance with this, transfection of AC16 cells with the anti-miR-146a inhibitor upregulated the expression of *IL-6* (1.3-fold, *P*<0.05 vs anti-miR+TNF-α), although only when the pro-inflammatory stimulus TNF-α was added to the medium (Fig. 4B). As previously reported (Palomer et al., 2009), treatment of AC16 cells with TNF-α (100 ng/ml for 24 h) significantly induced the expression of *IL-6*, *MCP-1* and *TNF-α*, regardless of miR-146a levels. After that, an EMSA was carried out to verify whether modulation of miR-146a levels in AC16 cells led to changes in NF-κB activity. As shown in Fig. 4C, NF-κB formed four specific DNA-binding complexes (I to IV) with nuclear proteins. The competitor lane confirmed that all four complexes were specific for the NF-κB probe, and supershift analyses provided evidence that only complexes I and II contained the p65 subunit of NF-κB. Interestingly, complexes I, II and III were increased in TNF-α-treated cells, but no variations were detected after miR-146a modulation. These results suggest that *IL-6* and *MCP-1* gene expression might be regulated by transcription factors other than NF-κB.

### Inhibition of AP-1 activity by miR-146a coincides with a reduction in MMP-9 activity

**Fig. 5.**
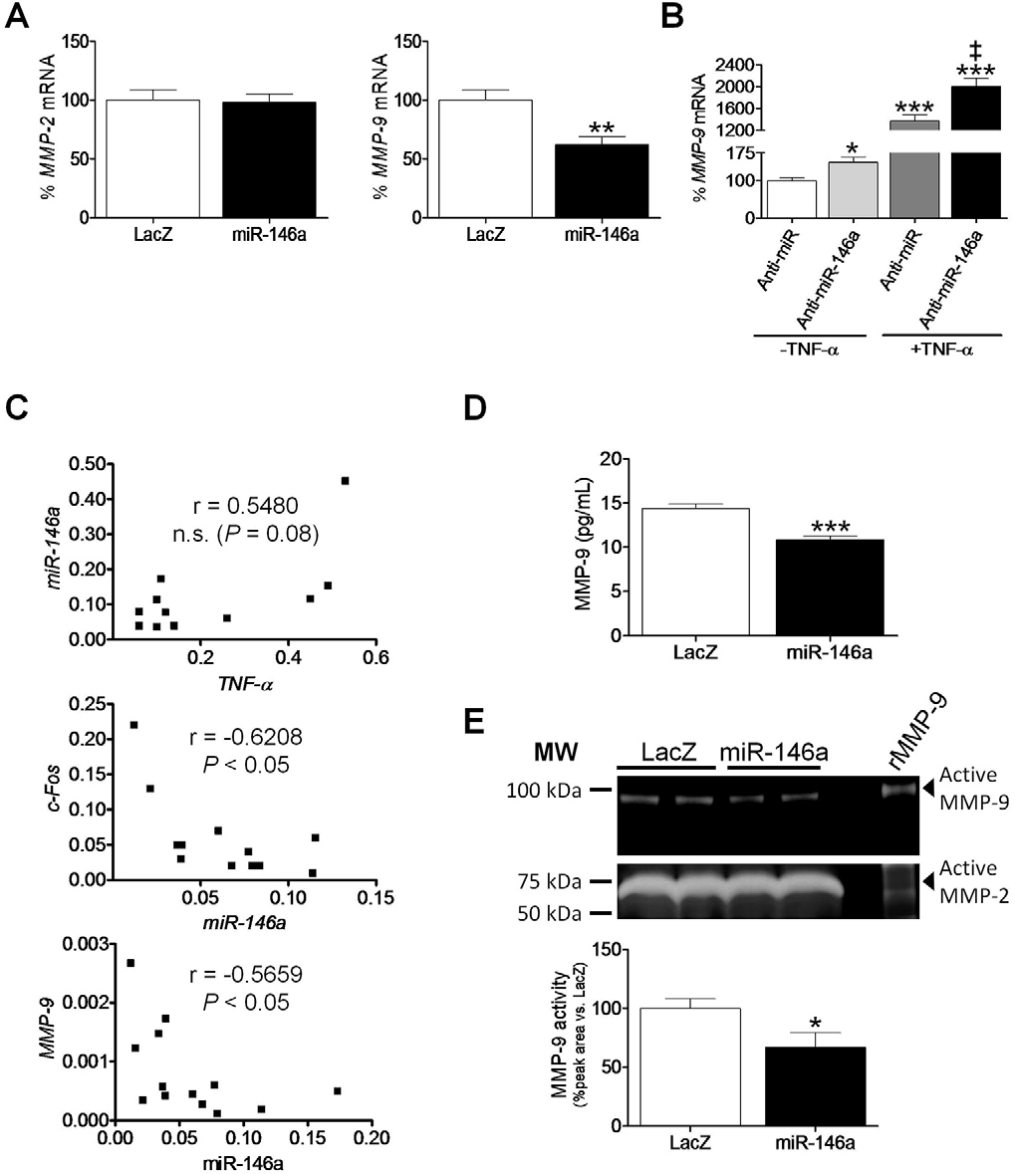
**miR-146a downregulates MMP-9 expression, secretion and activity in human cardiac cells.** Relative quantification of *MMP-2* and *MMP-9* mRNA levels in non-differentiated human cardiac AC16 cells transfected with: (A) *lacZ*- or miR-146a-carrying plasmids, or (B) a human anti-miR-146a inhibitor or an anti-miR negative control (Anti-miR). The graphs represent the quantification of 18S-normalized mRNA levels, expressed as a percentage of control samples ±s.d. (C) Spearman rank correlation between *TNF-α* and miR-146a, miR-146a and *Fos*, and miR-146a and *MMP-9* gene expression in left ventricular tissue obtained from patients undergoing heart transplantation. The relative transcript levels of the target genes, in arbitrary units, were used to calculate the Spearman correlation coefficients (n.s., non-significant). (D) Determination by ELISA of MMP-9 secretion into the culture media in AC16 cells transfected with *lacZ*- or miR-146a-carrying plasmids. (E) Representative gel zymography and corresponding densitometric analysis of MMP-9 gelatinolytic activity in culture media of cells transfected with *lacZ*- or miR-146a-carrying plasmids. MW, molecular weight; rMMP-9, human recombinant MMP-9. (A,D,E) *P<0.05, **P<0.01 and ***P<0.001 vs *lacZ*; (B) *P<0.05 and ***P<0.001 vs Anti-miR–TNF-α; ^‡^*P*<0.05 vs Anti-miR+TNF-α.

Examination of *MMP-9* expression in human cardiac cells transfected with miR-146a revealed that, unlike *MMP-2*, its transcript levels were downregulated (∼40% reduction, *P*<0.05, Fig. 5A). According to these results, anti-miR-146a stimulated *MMP-9* expression regardless of the presence (1.5-fold, *P*<0.001) or absence (1.5-fold, *P*<0.001) of TNF-α (Fig. 5B). We next examined this issue in left ventricular tissue obtained from patients undergoing heart transplantation. The relative expression of miR-146a positively correlated with that of *TNF-α* (Fig. 5C, top panel, Spearman rank correlation *r*=0.5480), a finding that, despite being only marginally significant (*P*=0.08), matched the previous results obtained in AC16 cells fairly closely. Of note, miR-146a levels negatively correlated with the expression of *Fos* (Fig. 5C, middle panel, Spearman rank correlation *r*=−0.6208, *P*<0.05) and *MMP-9* (Fig. 5C, bottom panel, *r*=−0.5659, *P*<0.05) in these patients. Finally, we aimed to examine the effects of *MMP-9* downregulation by miR-146a on its enzymatic activity. As expected, miR-146a overexpression elicited a reduction in MMP-9 secretion to the media (∼25% reduction, *P*<0.001, Fig. 5D) and MMP-9 activity (∼35% reduction, *P*<0.05, Fig. 5E). The zymogram also yielded a stronger gelatinolytic activity band, which was concentrated in the area corresponding to the molecular mass of MMP-2 (62 kDa), which was not modified under our conditions.

## DISCUSSION

The molecular mechanisms that lie behind the development of heart failure induced by cytokines remain, at least in part, elusive, but there are consistent observations indicating that chronic immune activation and anomalous miRNA expression come together in the failing heart (van de Vrie et al., 2011). Our results demonstrate that miR-146a is strongly induced in human cardiac cells and neonatal rat cardiomyocytes exposed to TNF-α *in vitro*, as well as in the heart of transgenic mice with cardiac-specific overexpression of TNF-α. This is not surprising, given that this miRNA has already been shown to be transcriptionally upregulated after NF-κB activation by TNF-α and IL-1β in other cell types (Taganov et al., 2006; Perry et al., 2008; Schroen and Heymans, 2012; Li et al., 2012). This regulatory mechanism is fundamental because continuous NF-κB activation aggravates cardiac remodeling, worsens cardiac function, and hastens progression to heart failure and cardiac hypertrophy (Bergman et al., 1999; Stetson et al., 2001). In agreement with this, miR-146a, which is abundantly expressed in the heart (van de Vrie et al., 2011), protects the myocardium from ischemia-reperfusion injury in a process that involves attenuation of NF-κB activation (Wang et al., 2013).

Owing to the crucial function of miRNAs in controlling mRNA expression, the discovery of currently unknown targets of specific miRNAs is paramount, but the fact that most miRNAs are only partially complementary to their target genes hinders this identification. Therefore, the chief finding of this study is the identification of a new miR-146a target, *Fos*, an AP-1 subunit that is rapidly activated upon cardiac stress to mediate changes in gene expression (Shieh et al., 2011). Validation of new miRNA targets is often complicated because many putative targets display little or indeed no detectable modification when tested *in vitro* (Small et al., 2010), and this might account for the relatively modest effect of miR-146a on *Fos* mRNA levels, despite the huge miR-146a changes observed in transfected cells. The lack of any change in Jun protein levels indicated the specificity of miR-146a over the Fos subunit of the AP-1 transcription factor.

The next logical step in this study consisted of finding out which genes were regulated by Fos–AP-1 under our conditions. AP-1 protein typically binds to TREs in target promoter regions often adjacent to NF-κB or nuclear factor of activated T-cells (NFAT) to coordinately regulate transcription in response to immune and inflammatory stimuli (Chinenov and Kerppola, 2001). Gene expression assessment revealed that miR-146a was modulating mRNA levels of *IL-6* and *MCP-1* in AC16 cells. Taking into account that this was not accompanied by changes in the DNA-binding activity of NF-κB, and bearing in mind that *in silico* data indicate that these pro-inflammatory genes are not direct targets of miR-146a, our results suggest that AP-1 might be regulating their expression. In support of this, we can cite our previous data demonstrating that, besides NF-κB, other transcription factors are controlling *IL-6* and *MCP-1* mRNA levels in human cardiac cells (Palomer et al., 2014; Álvarez-Guardia et al., 2011). In fact, other studies have demonstrated the involvement of AP-1 but not NF-κB in the transcriptional control of *IL-6* in other cell types (Lu et al., 2010). In contrast, the expression of other genes such as *ATF4*, *BiP/GRP78*, *IRAK1*, *IRAK2*, *TRAF2*, *TRAF6*, *ET-1*, *FN1*, lipin 1, *LPL*, *TGF-β1*, type I collagen or type IV collagen did not seem to be regulated by miR-146a. In fact, the expression of AP-1 target genes depends on the cell type and is also influenced by the particular signals that trigger their expression (Chinenov and Kerppola, 2001). Besides regulation at the transcriptional level, additional mechanisms might contribute to the target gene specificity of AP-1, including mRNA translation and turnover, post-translational modifications, selective dimerization, protein stability, and interactions with other regulatory proteins and transcription factors (Chinenov and Kerppola, 2001; Schonthaler et al., 2011).

Interstitial fibrosis is a characteristic pathological alteration of myocardial remodeling that occurs in several cardiomyopathies and is considered as a primary determinant of deteriorated performance of the heart. Fibrosis occurs as a result of excess deposition of extracellular matrix proteins in the myocardium (Pan et al., 2012). A complex interplay of transcription factors is implicated in the regulation of extracellular matrix protein homeostasis, including NF-κB and AP-1. In particular, the Fos–AP-1 pathway transcriptionally stimulates the synthesis of ET-1 and the deposition of collagen (type I, type IV collagens), fibronectin and TGF-β, thereby causing changes in the extracellular matrix that alter cardiac cell proliferation and function, ultimately leading to cardiomyocyte hypertrophy and heart failure (Pan et al., 2012; Wang et al., 2009; Avouac et al., 2012). Even so, miR-146a overexpression did not inhibit the expression of most cardiac-fibrosis-related genes examined in human cardiac cells, except for *MMP-9*. MMPs are under the transcriptional control of AP-1 (Takimoto and Kass, 2007) and NF-κB (Meiners et al., 2004), which are in turn induced by growth factors and inflammatory cytokines in cardiac cells, although MMP activity is also dependent on post-translational modifications. MMPs are the enzymes responsible for controlling extracellular matrix remodeling in the heart and, interestingly, inhibition of these enzymes is associated with reduced collagen deposition and lower cardiac fibrosis (Meiners et al., 2004). This astonishing paradox is due to the fact that total collagen amount in the heart depends on both the synthesis and degradation. The results reported here demonstrate that miR-146a can inhibit MMP-9 expression and activity in human cardiac cells. Infiltrating cells (i.e. neutrophils, macrophages and fibroblasts) together with cardiomyocytes are the major source of MMPs in the myocardium (Li et al., 2001). Macrophages are an important source of MMP-9 during acute myocardial infarction and, for instance, MMP-9-knockout mice show a reduced rupture rate and attenuated ventricular dilation during myocardial infarction (Fang et al., 2010). A recent study has also reported that miR-146a might be a potential inhibitor of MMP-9 secretion in macrophages, although regulation in these cells was achieved through attenuation of the inflammatory response by blocking the TRAF6-IRAK1 pathway (Yang et al., 2011). Likewise, cardiac fibroblasts express MMP-9 after treatment with TNF-α, a fact that is concomitant with a decrease in collagen synthesis (Brown et al., 2007). Therefore, all these results indicate that the miR-146a-mediated inhibition of MMP-9 might occur in both infiltrating cells and cardiomyocytes in the heart, by this means magnifying its beneficial effects.

A major drawback of this study is the origin of the AC16 cells, which were derived from the fusion of primary ventricular cells and SV-40-transformed fibroblasts. The heart consists of various cell types, including cardiomyocytes and cardiac fibroblasts, which play a pivotal role in cardiac development and function (Palomer et al., 2009). Both cell types are capable of secreting TNF-α and are responsive to the action of this cytokine (Turner et al., 2007) but, in diseased states, quiescent cardiac fibroblasts are transformed into myofibroblasts, becoming a major source of pro-inflammatory molecules (Brown et al., 2005), in addition to the main source of extracellular matrix production (Pan et al., 2012). As stated above, excess extracellular matrix production by activated cardiac fibroblasts during cardiac hypertrophy, heart failure and myocardial infarction promotes interstitial fibrosis. The consequent cardiac remodeling might eventually lead to functional decompensation and development of heart failure due to apoptosis of cardiac myofibroblasts (Camelliti et al., 2005).

### Conclusions

**Fig. 6.**
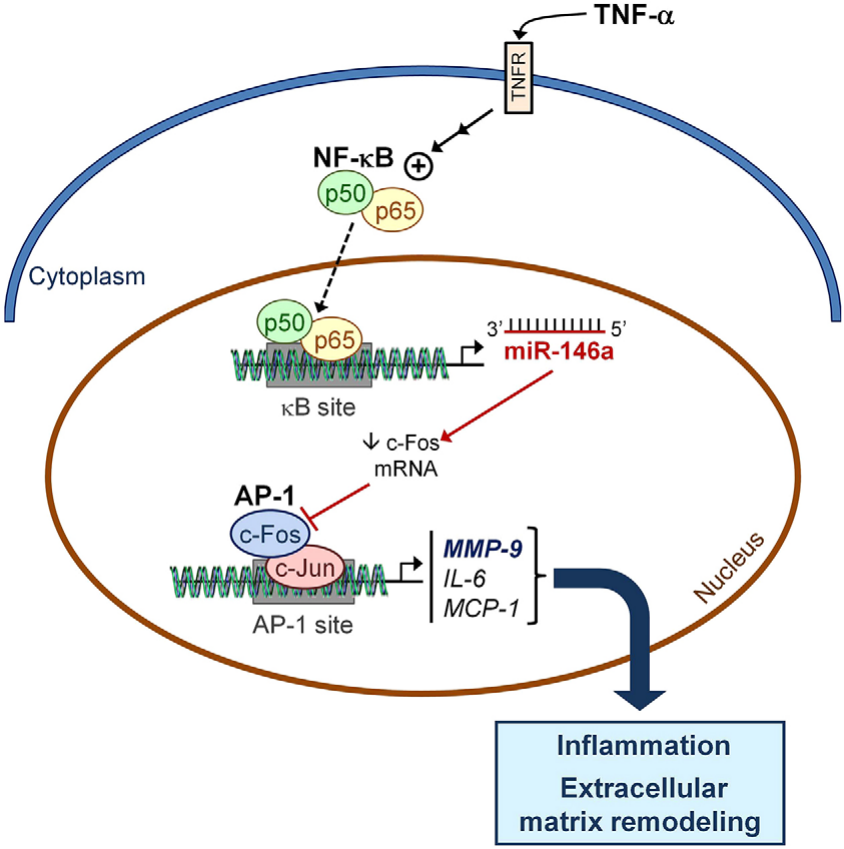
**Schematic model depicting the potential role of miR-146a in TNF-α-induced effects in the heart.** Exposure of cardiac cells to TNF-α strongly induces miR-146a, probably in a process dependent on NF-κB transcriptional activity (dashed arrow). Enhanced miR-146a levels are directly responsible for *Fos* expression downregulation. The subsequent reduction in AP-1 DNA-binding activity results in the modulation of inflammation by attenuating *IL-6* and *MCP-1* expression, together with a reduction in MMP-9 expression and activity. AP-1, activator protein-1; Fos, FBJ murine osteosarcoma viral oncogene homolog; IL-6, interleukin 6; MCP-1, monocyte chemoattractant protein 1; MMP-9, matrix metalloproteinase 9; NF-κB, nuclear factor-κB; TNF-α, tumor necrosis factor α.

In summary, the results reported here demonstrate that *Fos* is a direct target of miR-146a activity and that downregulation of the Fos–AP-1 pathway by miR-146a can inhibit MMP-9 activity (Fig. 6). Fos is one of the immediate early genes whose expression is boosted during ischemic injury, heart failure and cardiomyopathy. Likewise, it has been reported that *Fos* gene expression is stimulated as a result of insulin insufficiency in the diabetic myocardium (Wang et al., 1999) and also in the adipose tissue of streptozotocin-induced diabetic rats (Olson and Pessin, 1994). In fact, the transcriptional activity of AP-1 is among the most robustly enhanced of 54 transcription factors examined in the failing heart (Freire et al., 2007). In order to prevent the pathological effects caused by its dysfunction, regulation of AP-1 is complex and occurs at multiple interwoven transcriptional and post-transcriptional levels. This includes transcription of its subunits, mRNA translation and turnover, protein stability and activity, subcellular localization, and interaction with other transcription factors and cofactors (Schonthaler et al., 2011). Here, we demonstrate that miR-146a might post-transcriptionally regulate Fos levels and, consequently, AP-1 activity as well. Furthermore, the results presented here are very appealing because numerous studies have shown that upregulation of *MMP-2* and *MMP-9* expression correlates fairly well with heart failure, whereas their inhibition suppresses ventricular remodeling, myocardial dysfunction and development of heart failure (Meiners et al., 2004). In recent years, the development of antisense-oligonucleotide-mediated (anti-miR) knockdown and miRNA overexpression techniques has become a very attractive pharmacological target in the treatment of cardiovascular disease. In this respect, miR-146a emerges as a new and promising therapeutic tool for preventing cardiac disorders associated with inflammatory states in the heart.

## MATERIALS AND METHODS

### Cell culture and transfection

The human AC16 cell line, which develops many of the biochemical and morphological properties characteristic of cardiac muscle cells, even though it does not form completely differentiated cardiomyocytes, was grown as previously described (Davidson et al., 2005). Briefly, non-differentiated AC16 cells were maintained in medium composed of Dulbecco's modified Eagle's medium (DMEM):F12 (Life Technologies, Spain) supplemented with 12.5% fetal bovine serum (FBS), 1% penicillin-streptomycin and 1% Fungizone (Life Technologies), and grown at 37°C in a humid atmosphere of 5% CO_2_/95% air until they reached 70-80% confluence. For *in vitro* overexpression studies, AC16 cells were transfected with pcDNA3/pre-miR-146a (Addgene plasmid #15092) (Taganov et al., 2006) and the corresponding *lacZ*-carrying plasmid (Life Technologies) as a control. Cells were transfected for 48 h with Lipofectamine 2000 in OPTI-MEM reduced serum medium following the manufacturer's recommendations (Life Technologies). Transfection time and the DNA to Lipofectamine ratio were set after optimization with the corresponding *lacZ*-carrying plasmid and using a β-galactosidase reporter gene staining kit (Sigma-Aldrich Co. LLC., St Louis, MO, USA). Downregulation of miR-146a activity was carried out by transfecting AC16 cells with 50 nmol/l human anti-miR-146a inhibitor, using a random sequence anti-miR molecule as negative control (Life Technologies).

To obtain neonatal rat cardiomyocytes, 1- to 2-day-old Sprague-Dawley rats were decapitated and their hearts removed. Hearts were digested with a collagenase solution (Collagenase Type I, Life Technologies) followed by differential plating. Cells were plated at a density of 2.5×10^4^ cells/well in six-well plates coated with 1% gelatin, and cultured overnight in plating medium [DMEM supplemented with 10% horse serum, 5% newborn calf serum, 50 mg/l gentamicin and 10 mM cytosine b-D-arabino furanoside (Ara C)]. Ara C was added to suppress the growth of the remaining fibroblasts. Sixteen hours after isolating cells, neonatal rat cardiomyocytes were incubated in serum-free medium consisting of DMEM and gentamicin (50 mg/l) as the sole substrate for 24 h. Thereafter, the medium was replaced with experimental medium consisting of serum-free medium enriched with 0.25 mM L-carnitine, 0.25 mU/ml insulin and 1% bovine serum albumin. All procedures were approved by the University of Barcelona Bioethics Committee, as stated in Law 5/21 July 1995 passed by the Generalitat de Catalunya.

After treatment, RNA and protein were extracted from cardiac cells as described below. Culture supernatants were collected, and secretion of MMP-9 was assessed by enzyme-linked immunosorbent assay (Life Technologies).

### TNF-α transgenic mouse cardiac sample preparation

We used transgenic TNF1.6 male mice (8- to 12-weeks old) with cardiac-specific overexpression of TNF-α, which has been established as a suitable model of cytokine-induced cardiomyopathy and congestive heart failure (Kubota et al., 1997). These transgenic mice develop myocardial inflammation with premature death from heart failure in association with extracellular matrix remodeling (Kubota et al., 1997; Li et al., 2000). Left ventricular end-diastolic diameter and left ventricular end-diastolic pressure are significantly greater, and fractional shortening is significantly less in TNF1.6 than in wild-type mice (Matsusaka et al., 2005). Myocyte cross-sectional area and collagen volume fraction are also enhanced in the transgenic TNF1.6 mice compared with littermate controls (Matsusaka et al., 2005).

Mice were housed under standard light-dark cycle (12-h light/dark cycle) and temperature (21±1°C) conditions, and food and water were provided *ad libitum*. Ventricular sample tissues were obtained from mice euthanized using deep isoflurane (5%) anesthesia, rinsed in ice-cold phosphate buffer saline and snap-frozen in liquid nitrogen, as described previously (Álvarez-Guardia et al., 2010). The study was approved by the Institutional Animal Care and Use Committee of Thomas Jefferson University and conformed to the *Guide for the Care and Use of Laboratory Animals* published by the US National Institutes of Health (NIH Publication No. 85-23, revised 1996).

### Tissue collection

Left ventricular tissue was obtained from patients undergoing heart transplantation at the Hospital de la Santa Creu i Sant Pau, Barcelona. All participants provided informed consent. Transmural tissue samples (near the apex) were collected from patients (eight male and five female) with both dilated (DCM; *n*=10) and ischemic (ICM; *n*=3) cardiomyopathy. Samples were quickly frozen in liquid nitrogen in the operating room and stored at −80°C until processing for gene expression analysis. All the procedures were approved by the Reviewer Institutional Committee on Human Research of the Hospital de la Santa Creu i Sant Pau and conformed to the Declaration of Helsinki.

### RNA preparation and analysis

Total RNA was isolated using Ultraspec reagent (Biotecx, Houston, TX, USA). RNA samples were cleaned (NucleoSpin RNA II; Macherey-Nagel, Düren, Germany) and checked for integrity by agarose gel electrophoresis. The total RNA isolated by this method was undegraded and free of protein and DNA contamination. Relative levels of specific mRNAs were assessed by real-time reverse transcription-polymerase chain reaction (RT-PCR), as previously described (Palomer et al., 2014). Reverse transcription was performed from 0.5 µg total RNA using Oligo(dT)_23_ and M-MLV Reverse Transcriptase (Life Technologies). The PCR reaction contained 10 ng of reverse-transcribed RNA, 2X IQ™ SYBRGreen Supermix (Bio-Rad, Barcelona, Spain) and 900 nM of each primer. PCR assays were performed on a MiniOpticon™ Real-Time PCR system (Bio-Rad). Thermal cycling conditions were as follows: activation of Taq DNA polymerase at 95°C for 10 min, followed by 40 cycles of amplification at 95°C for 15 s and at 60°C for 1 min. The sequences of the forward and reverse primers used for amplification are shown in supplementary material Table S1. Optimal primer amplification efficiency for each primer set was assessed and a dissociation protocol was carried out to ensure a single PCR product. The results for the expression of specific mRNAs are always presented relative to the expression of the control gene.

To quantify the abundance of selected mature miRNAs, total miRNAs were reverse-transcribed using the Megaplex Primer Pools (Human Pool A v2.1 and Rodent Pool A) and the Taqman MicroRNA Reverse Transcription according to the manufacturer's instructions (Life Technologies). The RT reaction product was combined with 1 μl Taqman miRNA assay and 10 μl Taqman Universal PCR Master Mix No AmpErase UNG (Life Technologies) to a final volume of 20 μl. The quantitative real-time RT-PCR reaction was carried out on a MiniOpticon™ Real-Time PCR system at 95°C for 10 min, followed by 40 cycles at 95°C for 15 s and 60°C for 1 min. All samples were run in duplicates. *U6sRNA* expression was used for normalization purposes.

### Immunoblot analysis

To obtain total protein extracts, AC16 cardiac cells or frozen tissue slides were lysed in cold RIPA buffer with phosphatase and protease inhibitors (0.2 mmol/l phenylmethylsulfonyl fluoride, 1 mmol/l sodium orthovanadate, 5.4 µg/ml aprotinin). The homogenate was then centrifuged at 10,000 ***g*** for 30 min at 4°C, and the supernatant protein concentration was determined using the Bradford method (Bradford, 1976). Isolation of cytosolic and nuclear fractions was adapted from a previously described method (Ba et al., 2010). Briefly, AC16 cells were incubated on ice for 30 min in buffer A (10 mmol/l HEPES, pH 7.9, 10 mmol/l KCl, 0.2 mmol/l EDTA, 1 mmol/l dithiothreitol, plus phosphatase and protease inhibitors) containing 0.625% (v:v) Nonidet P-40. Cell lysates were centrifuged at 4°C, 10,000 ***g*** for 1 min, and supernatants were stored as cytosolic fraction. Pellets were suspended in buffer B (20 mmol/l HEPES, pH 7.9, 0.42 mol/l NaCl, 2 mmol/l EDTA and 1 mmol/l dithiothreitol, with phosphatase and protease inhibitors), centrifuged at 4°C, 13,000 ***g*** for 5 min, and the resultant supernatant (nuclear extract) stored at −80°C.

Proteins from whole-cell lysates and cytosolic/nuclear extracts were separated by sodium dodecyl sulfate-polyacrylamide gel electrophoresis (SDS-PAGE) on 10% separation gels and transferred to Immobilon polyvinylidene difluoride membranes (Millipore, Bedford, MA, USA). Proteins were detected using the Western Lightning^®^ Plus-ECL chemiluminescence kit (PerkinElmer, Waltham, MA, USA) and their size was estimated using protein molecular mass standards (Life Technologies). All antibodies were purchased from Santa Cruz Biotechnology (Inc., Heidelberg, Germany), except Actin (Sigma-Aldrich Co. LLC.).

### Electrophoretic mobility shift assay

The EMSA was performed using double-stranded oligonucleotides for the consensus binding sites of AP-1 and NF-κB (Santa Cruz Biotechnology). Nuclear extracts (NEs) from AC16 cells were isolated as previously reported (Palomer et al., 2011). Oligonucleotides were labeled by incubating the following reaction at 37°C for 2 h:2 µl oligonucleotide (1.75 pmol/µl), 2 µl of 5× kinase buffer, 1 µl of T4 polynucleotide kinase (10 U/µl) and 2.5 µl [γ-^32^P] ATP (3000 Ci/mmol at 10 mCi/ml, PerkinElmer, Waltham, MA, USA). The reaction was stopped by adding 90 µl of TE buffer (10 mmol/l Tris-HCl, pH 7.4, and 1 mmol/l EDTA). To separate the labeled probe from the unbound ATP, the reaction mixture was eluted in a Nick column (GE Healthcare Life Sciences, Barcelona, Spain) according to the manufacturer's instructions. Five micrograms of crude nuclear protein was incubated for 10 min on ice in binding buffer [10 mmol/l Tris-HCl, pH 8.0, 25 mmol/l KCl, 0.5 mmol/l dithiothreitol, 0.1 mmol/l EDTA, pH 8.0, 5% (v:v) glycerol, 5 mg/ml BSA and 50 µg/ml poly(dI-dC)] in a final volume of 15 µl. Then, specific competitor oligonucleotide or antibody for supershift assays were added and incubated for 15 min on ice. Subsequently, the labeled probe (100,000 cpm) was added and the reaction was incubated for an additional 15 min on ice. Finally, protein-DNA complexes were resolved by electrophoresis at 4°C on 5% (w:v) polyacrylamide gels in 0.5× Tris-borate-EDTA buffer and subjected to autoradiography.

### Gelatinase activity assay

MMP-9 activity was examined by gelatin zymography as previously reported in AC16 cell cultures after protein concentration using 3000 MW Amicon Ultra centrifugal filters (Millipore) (Casals et al., 2013). 150 μg of protein per lane were subjected to 10% SDS-PAGE electrophoresis (125 V for 90 min) using 0.2% gelatin-containing gels. After electrophoresis, gels were washed and incubated for 30 min at room temperature in Renaturing Buffer (Novex, Life Technologies) to remove the SDS. After this, gels were incubated with gentle agitation for 30 min with Developing Buffer (Life Technologies), rinsed three times with deionized water and stained by adding SimplyBlue Safe Stain (Life Technologies) for 1 h. 50 pg recombinant human MMP-9 (Life Technologies) were also run in parallel as a positive control for enzymatic activity. Proteolytic bands of 92 kDa, which correspond to the active form of MMP-9, were scanned and the intensity of the bands analyzed.

### Statistical analysis

Results are expressed as the mean±s.d. of three independent experiments for the *in vitro* studies, each consisting of three culture plates (*n*=9), and of five mice for the *in vivo* experiments. Significant differences were established by either the Student's *t*-test or one-way ANOVA, according to the number of groups compared, using GraphPad Prism software (GraphPad Software Inc. V4.03, San Diego, CA, USA). When significant variations were found by one-way ANOVA, the Tukey-Kramer multiple comparison post-test was performed. The non-parametric Spearman rank correlation coefficient was used to calculate the correlation between *Fos*, *miR-146a* and *TNF-α* expression. Differences were considered significant at *P*<0.05.

## Supplementary Material

Supplementary Material
