# Supplementary material for: miR-146a targets Fos expression in human cardiac cells
Source: Dis Model Mech. 2015 Sep 1;8(9):1081–91. doi: 10.1242/dmm.020768 (PMC4582106; doi:10.1242/dmm.020768)
Supplement: Supplementary Material [file supp_8_9_1081__index.html]

Supplementary Material 

# miR-146a targets *c-Fos* expression in human cardiac cells

## DMM020768 Supplementary Material

- Supplementary Material
