# Supplementary material for: miR-146a targets Fos expression in human cardiac cells
Source: Dis Model Mech. 2015 Sep 1;8(9):1081–91. doi: 10.1242/dmm.020768 (PMC4582106; doi:10.1242/dmm.020768)
Supplement: Supplementary Material [file supp_020768_DMM020768supp.pdf]

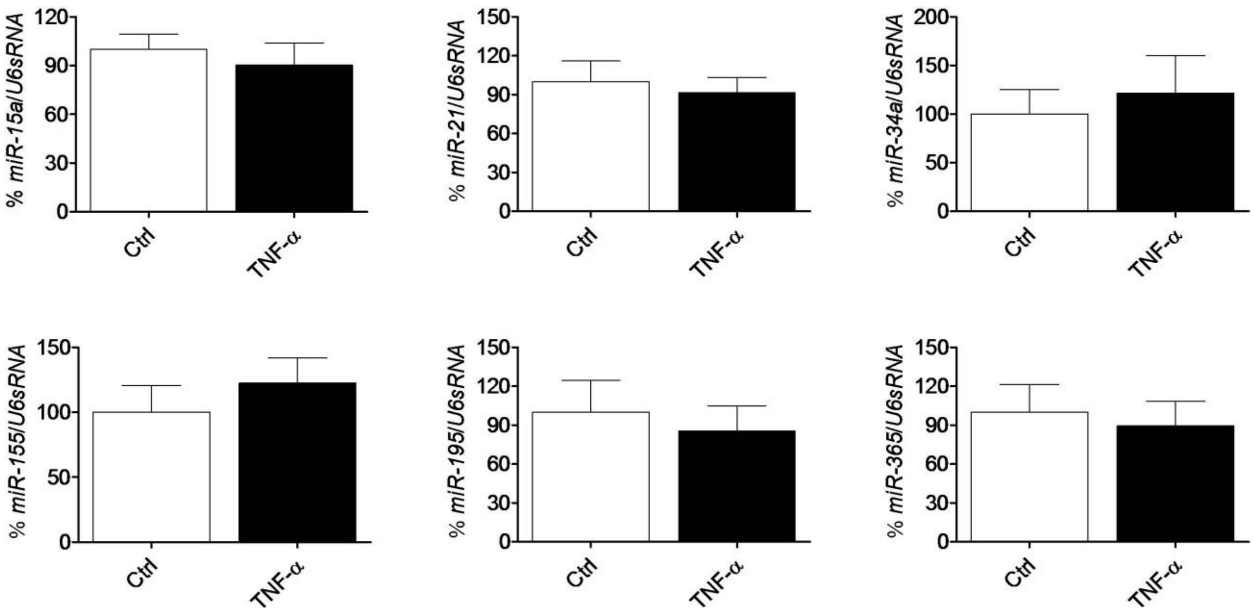

Fig. S1. Relative quantification of miR-15a, miR-21a, miR-34a, miR-155, miR-195 and miR-365 mRNA levels in samples obtained from non-differentiated AC16 cells treated with TNF-α (100 ng/ml, 24h). The graph represents the quantification of *U6sRNA*-normalized mRNA levels, expressed as a percentage of control samples ±SD.

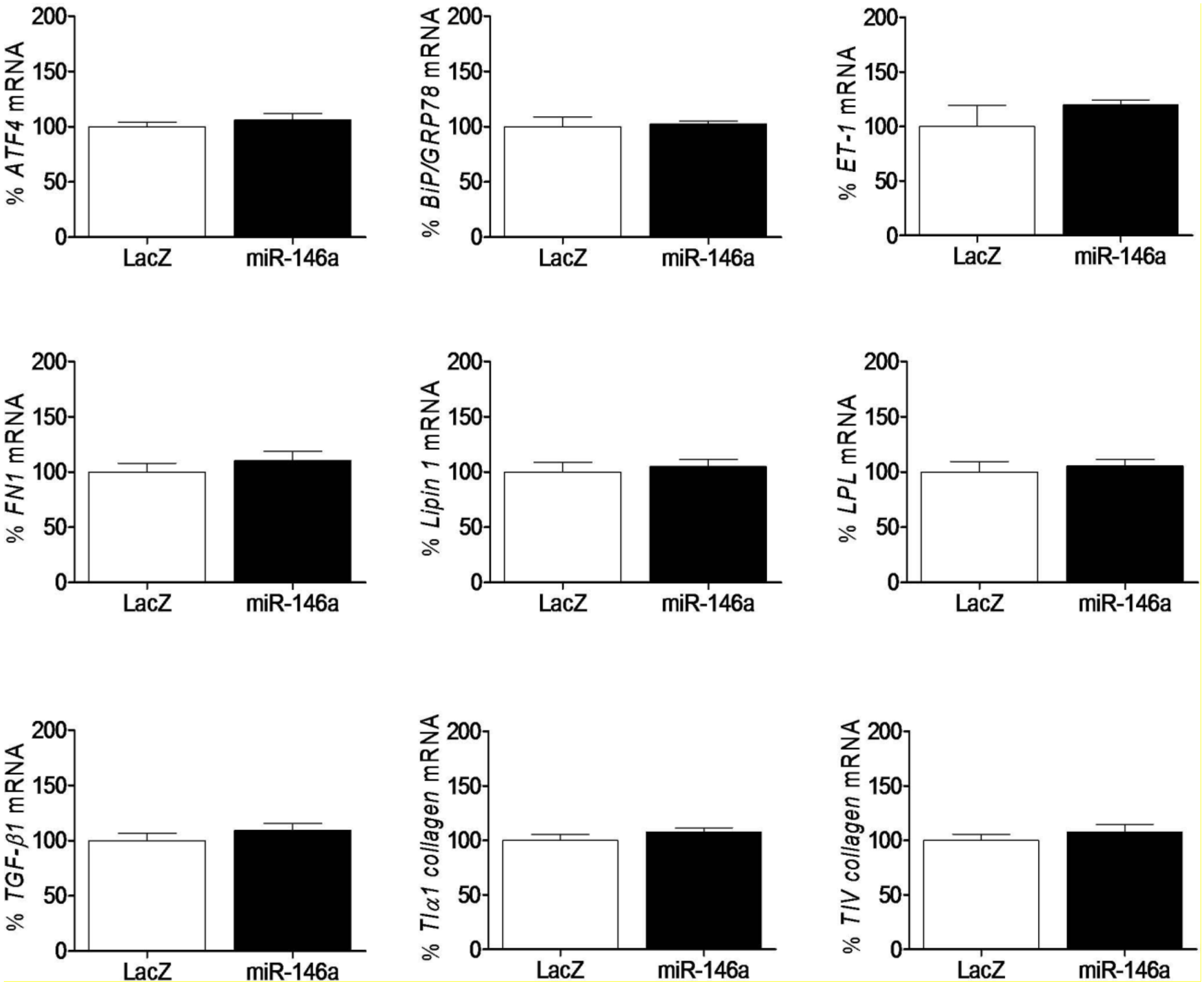

Fig. S2. Relative quantification of *ATF4*, *BiP/GRP78*, *endothelin 1 (ET-1)*, *fibronectin 1 (FN1)*, *lipin 1*, *lipoprotein lipase* (*LPL*), *TGF-β1*, *type I collagen* and *type IV collagen* mRNA levels in samples obtained from non-differentiated human cardiac AC16 cells transfected with LacZ- or pre-miR-146a-carrying plasmids. The graph represents the quantification of *18S*-normalized mRNA levels, expressed as a percentage of control samples ±SD.

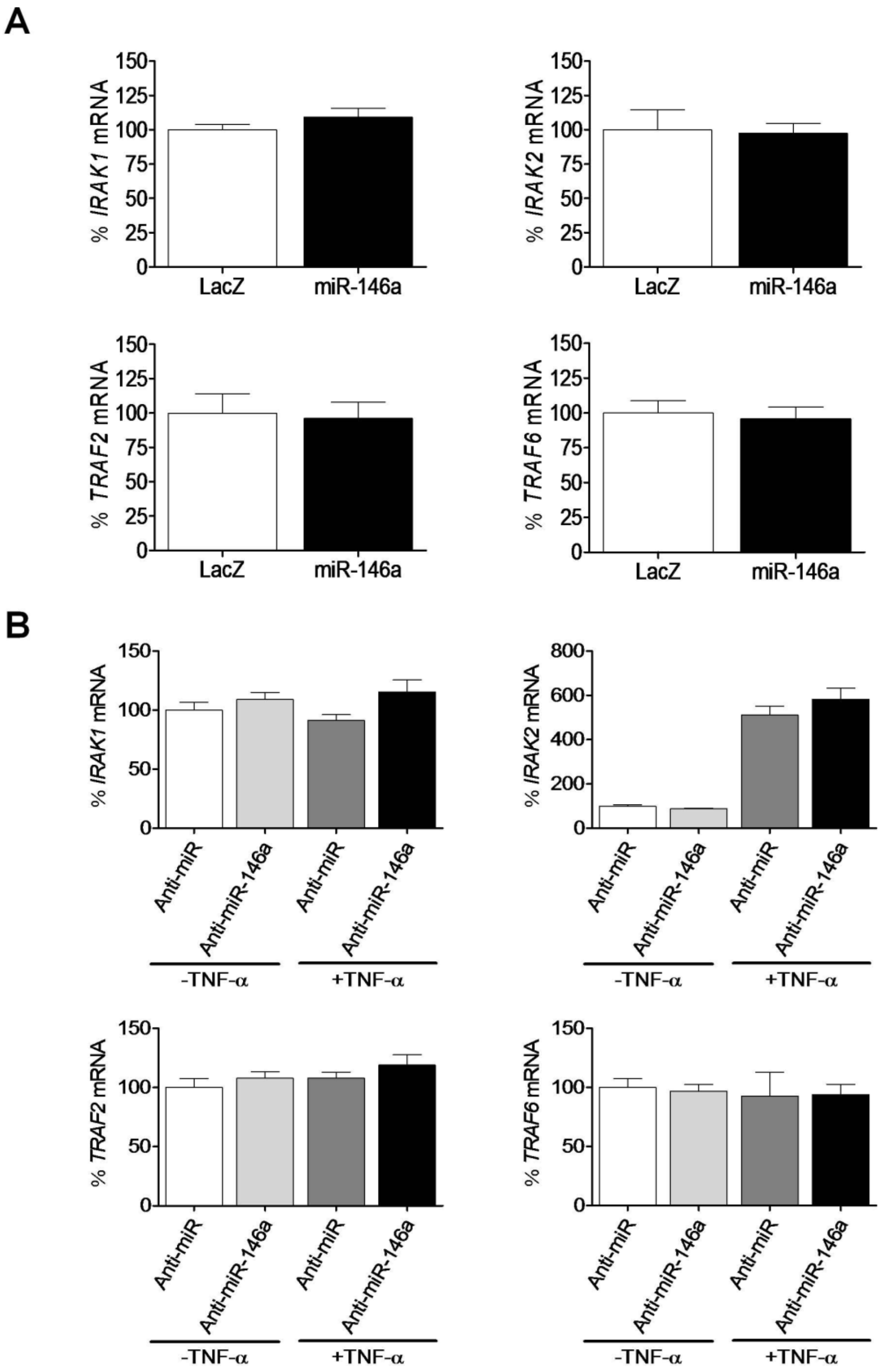

Fig. S3. Relative quantification of *IRAK1*, *IRAK2*, *TRAF2* and *TRAF6* mRNA levels in samples obtained from non-differentiated human cardiac AC16 cells transfected with: (**A**) LacZ- or pre-miR-146a-carrying plasmids, or (**B**) a human anti-miR-146a inhibitor or an anti-miR negative control. The graph represents the quantification of *18S*-normalized mRNA levels, expressed as a percentage of control samples ±SD.

Table S1. Primers used for the real-time RT-PCR reactions

Gene	Forward primers	Reverse primers
**Human**		
18S	5’-GCCGCTAGAGGTGAAATTCTTG-3’	5’-CATTCTTGGCAAATGCTTTCG-3’
ATF4	5’-GCGGGCTCCTCCGAAT-3’	5’-ATCCTCCTTGCTGTTGTTGGA-3’
BiP/GRP78	5’-ACTATTGCTGGCCTAAATGTTATGAG-3’	5’-TTATCCAGGCCATAAGCAATAGC-3’
c-Fos	5’-GGGATAGCCTCTCTTACTACCACTCA-3’	5’-GAAGTCCTGCGCGTTGACA-3’
ET-1	5’-TGCCACCTGGACATCATTTG-3’	5’-TGGACCTAGGGCTTCCAAGTC-3’
FN1	5’-TGGACCAAGTTGATGACACC-3’	5’-CACCAGGTTGCAAGTCACTG-3’
IL-6	5’-CCCCCAGGAGAAGATTCCAA-3’	5’-TCAATTCGTTCTGAAGAGGTGAGT-3’
IRAK1	5’-TGATGAGGACACGGTGTATGC-3’	5’-GCTCCACCTCGGTCAGGAA-3’
IRAK2	5’-GGGCATCCCTGCAATGG-3’	5’-TGGAATATCACTGAGGAGTAAGTCCTT-3’
Lipin 1	5’-CAGACAGCAAACAAGACGGATTC-3’	5’-CGCCGTCAGCACCAAGAT-3’
LPL	5’-CCAAACTGGTGGGACAGGAT-3’	5’-ATGGACATTGTCCAGAGGGTAGTTA-3’
MCP-1	5’-GCTGTGATCTTCAAGACCATTGTG-3’	5’-TGGAATCCTGAACCCACTTCTG-3’
MMP-2	5’-TTGATGGCATCGCTCAGATC-3’	5’-TGTCACGTGGCGTCACAGT-3’
MMP-9	5’-CCACCACAACATCACCTATTGG-3’	5’-GAGGCGCGGGCAAA-3’
TGF-β1	5’-AACCCACAACGAAATCTATGAC-3’	5’-GAGGTATCGCCAGGAATTGT-3’
TNF-α	5’-TCTTCTCGAACCCCGAGTGA-3’	5’-GGAGCTGCCCCTCAGCTT-3’
TRAF2	5’-TTGAAGCCCTGAGTAGCAAGGT-3’	5’-CCATCGCCAGGTCCTTGA-3’
TRAF6	5’-TGCCCTACAGCCCCAATTC-3’	5’-GCCAAGTGATTCCTCTGCATCT-3’
TI1α collagen	5’-GAACGCGTGTCATCCCTTGT-3’	5’-GAACGAGGTAGTCTTTCAGCAACA-3’
TIV collagen	5’-ACTCTTTTGTGATGCACACCA-3’	5’-AAGCTGTAAGCGTTTGCGTA-3’

**Mice**		
APRT	5’-CAGCGGCAAGATCGACTACA-3’	5’-AGCTAGGGAAGGGCCAAACA-3’
MMP-9	5’-CCTGGAACTCACACGACATCTTC-3’	5’-TGGAAACTCACACGCCAGAA-3’
**Rat**		
APRT	5’-CCCACTGTATCAGCCTCCTATTCT-3’	5’-CCCGGTTCTAAGGCATCTTTC-3’
c-Fos	5’-GACAGCCTTTCCTACTACCATTCC-3’	5’-CGCAAAAGTCCTGTGTGTTGA-3’
MMP-9	5’-GAGGATCCGCAGTCCAAGAA-3’	5’-GCACCGTCTGGCCTGTGTA-3’

Abbreviations: ATF4, activating transcription factor 4; BiP/GRP78, binding immunoglobulin protein/glucose-regulated protein 78; c-Fos, FBJ murine osteosarcoma viral oncogene homolog; ET-1, endothelin 1; FN1, fibronectin 1; IL-6, interleukin 6; IRAK, interleukin-1 receptor-associated kinase; LPL, lipoprotein lipase; MCP-1, monocyte chemoattractant protein 1; MMP, matrix metalloproteinase; TGF-β1, transforming growth factor β; TNF-α, tumor necrosis factor α; TRAF, TNF receptor-associated factor 2; TI1α collagen, type 1α collagen; TIV collagen, type IV collagen.
